# Development of myelin in fetal and postnatal neocortex of the pig, the European wild boar *Sus scrofa*

**DOI:** 10.1007/s00429-023-02633-y

**Published:** 2023-03-31

**Authors:** Eric Sobierajski, German Lauer, Katrin Czubay, Hannah Grabietz, Christa Beemelmans, Christoph Beemelmans, Gundela Meyer, Petra Wahle

**Affiliations:** 1 Faculty of Biology and Biotechnology, Developmental Neurobiology, Ruhr University Bochum, 44870 Bochum, Germany; 2 Regionalverband Ruhr Grün, Forsthof Üfter Mark, Forsthausweg 306, 46514 Schermbeck, Germany; 3 Department of Basic Medical Science, Faculty of Medicine, University of La Laguna, 38200 Santa Cruz de Tenerife, Tenerife Spain

**Keywords:** Myelin basic protein, Myelin-associated protein, Olig2, GFAP, Vimentin, Parvalbumin

## Abstract

Myelination of the neocortex of altricial species is mostly a postnatal event, and the appearance of myelin has been associated with the end of the critical period for ocular dominance plasticity in rodent visual cortex. Due to their precocality, ungulates may tell a different story. Here, we analyzed the development of PDGFRα positive oligodendrocyte precursor cells and expression of myelin proteins in the laminar compartments of fetal and postnatal porcine cortex from E45 onwards. Precursor cell density initially increased and then decreased but remained present at P90. MAG and MBP staining were detectable at E70 in subventricular zone and deep white matter, ascending into gyral white matter at E85, and into the gray matter and marginal zone at E100 (birth in pig at E114). Protein blots confirmed the declining expression of PDGFRα from E65 onwards, and the increase of MBP and MAG expression from E80 onwards. Somatosensory input elicited by spontaneous activity is considered important for the formation of the body representation. Indeed, PDGFRα, MBP and MAG expression started earlier in somatosensory than in visual cortex. Taken together, myelination proceeded in white and gray matter and marginal zone of pig cortex before birth with an areal-specific time course, and an almost mature pattern was present at P5 in visual cortex.

**Supplementary Information:**

The online version contains supplementary material available at 10.1007/s00429-023-02633-y.

## Introduction

Oligodendrocytes form the myelin sheaths of axons of CNS neurons and deliver metabolic and trophic support for neurons (for review see Nave and Werner 2014; Sampiao-Batista and Johansen-Berg 2017). Guided by chemoattractants, oligodendrocyte progenitor cells (OPC) invade the cortex from the ganglionic eminences and, later, originate from the subventricular zone. The latter cohort delivers most oligodendrocytes for the adult cortex (Kessaris et al. 2006). In the adult, GFAP + stem cells in the subventricular zone generate oligodendrocytes (Menn et al. 2006; Huang et al. 2020). OPC proliferate locally, the progeny develops into pre-myelinating oligodendrocytes which mature into myelinating oligodendrocytes. The developmental onset of myelination has been shown to depend on the electrical activity of axons (Demerens et al. 1996) and diffusible factors. Platelet-derived growth factor (PDGF) via PDGF receptor alpha (PDGFRα) and Notch signaling as well as neurotransmitter receptor signaling and communication with astrocytes support survival and proliferation of precursors and slow the pace of maturation (Noble et al. 1988; Mangin et al. 2012; Spampinato et al. 2014). With further differentiation, young oligodendrocytes lose the expression of PDGFRα, and survival becomes dependent on contact with axons (Barres et al. 1992; Barres and Raff 1999). ATP released from electrically active axons recruits astrocytes to dampen the proliferative drive of precursors and promote formation of myelination sheaths (Ishibashi et al. 2006).

In laboratory rodent telencephalon, OPC proliferate massively until postnatal day (P) 11 followed by the period of myelin deposition and compactation up to P60 (Matthieu et al. 1973). Myelin becomes detectable postnatally with single myelinated fibers in internal capsule, cingulate bundle, and cortical white matter. Around P14, myelin occurs in gray matter and corpus callosum. The newly formed myelin sheaths undergo a compactation, which involves myelin basic protein (MBP), one major myelin component. MBP production is upregulated by integrins as well as action potential-triggered glutamate release (Laursen et al. 2011; Wake et al. 2011). Myelin reaches near-adult level around the third postnatal week and continues to increase until the third postnatal month (Jacobson 1963; Bjelke and Seiger 1989; Mengler et al. 2014). The presence of myelin and the expression of myelin inhibitory proteins such as MAG as a ligand for Nogo receptors have been correlated with the closure of the critical period of sensory plasticity in rodent visual cortex (McGee et al. 2005; Jitsuki et al. 2016). However, developmental and adult white matter plasticity such as regulation by electrical activity, sensory experience, age, diet and lifestyle are still incompletely understood (Horton and Hocking 1997; Sampaio-Baptista and Johansen-Berg 2017).

The pig has gained importance as a translational model for neurodevelopmental as well as neurodegenerative and neuroinflammatory disorders. Most of the latter are known to impair myelination, and encephalomyelitic demyelination has been modeled in pig (Singer et al. 2000). Pig with its gyrencephal cortex has been reported to resemble human in the proportion of white matter (Stinnett et al. 2017) which, in human, comprised about half of the adult brain volume (Sampiao-Batista and Johansen-Berg 2017). As in human, myelination in pig cortex starts prenatally (Vallet and Miles 2012; Tolcos et al. 2011), reporting for instance myelin basic protein expression at E100 in parietal cortex of domestic Large-white pig fetuses (Kalanjati et al. 2017). However, a detailed analysis of cortical myelination in hoofed animals is lacking. This prompted the present study. Here, we report a massive proliferation of PDGFRα positive OPC before midgestation. Myelin protein expression starts prenatally with somatosensory preceding visual cortex. An almost mature pattern of cortical myelination in visual cortex is established perinatally.

## Materials and methods

### Animal material

The material for the immunohistochemical staining is from our fetal pig brain collection (Ernst et al. 2018; Sobierajski et al. 2022). Material has been obtained from the Üfter Mark area managed by the Regionalverband Ruhr Grün. Fetuses derived from mostly young (first pregnancy) sows and piglets individually hunted for population control (not for research) in accordance with the German Game Law or killed in road accidents. Law requests disposal of viscera including sexual organs. During evisceration, the uteri were examined for pregnancies. For histology, fetuses were extracted from the fetal membranes at the Forsthof Üfter Mark and immersed in cold 4% paraformaldehyde (PFA) in 0.1 M phosphate buffer pH 7.4. For Western blots, fetuses were immersed in cold Ringer solution. The P5 domestic German Landrace piglet was donated by the Institutes of Physiology and Anatomy, Medical Faculty, University Mannheim (donated by Prof. Martin Schmelz and Prof. Dr. Maren Engelhardt). Material was transported to Ruhr University within less than 24 h postmortem (Table 1). Animals, body organ weight and brains were documented. Brains were dissected, meninges removed and the cortex was cut into slabs.

**Table 1:** Pig material

Fetal age (gestation)	Date of kill	Litter size, sex	Time 1 [h]	Time 2 [h]	Method
E45 (0.40)	06. 06. 2015	4 (3m, 1f)	1.5	4 days	IHC, IF
E60 (0.53)	25. 06. 2015	5 (3m, 2f)	1	13	IHC, IF
E65 (0.57)	31. 01. 2022	4 (1m, 3f)	n. d	4.5	WB
E70 (0.61)	08. 01. 2016	6 (4m, 2f)	4	10	IHC, IF
E80 (0.70)	15. 11. 2018	3 (2m, 1f)	11	4.5	WB
E85 (0.75)	22. 01. 2016	7 (2m, 5f)	1.5	10	IHC, IF
E95 (0.83)	15. 11. 2018	3 (2m, 1f)	11	4.5	WB
E95 (0.83)	03. 12. 2018	3 (2m, 1f)	11	4.5	WB
E100 (0.88)	21. 06. 2015	5 (3m, 2f)	1.5	19	IHC, IF
E100 (0.88)	03. 12. 2018	2 (1m, 1f)	3	6	WB
E110 (0.96)	10. 03. 2017	7 (4m, 3f)	2	19	IHC, IF
E110 (0.96)	03. 12. 2018	2 (f)	3	6	WB
P5, domestic	12. 02. 2018	n.d	n.a	2	IHC, IF
P30	08. 03. 2016	1 (m)	0.1	1	IHC, IF
P90	16. 06. 2019	2 (f)	2	16	IHC, IF, WB

Gestation, proportional age with birth at E114 set to 1; f, female; m, male; n.a., not applicable; n.d., not determined. Time 1 is the period until the fetuses were removed from the amniotic membranes and immersed into fixative. Time 2 is the period for retrieving the fetuses, transport to the lab, documentation, and dissection. For E45, time 2 is the time for fixation in toto before dissection of the embryos. *IHC* immunohistochemistry, *IF* immunofluorescence, *WB* western blot

**Fig. 1:**
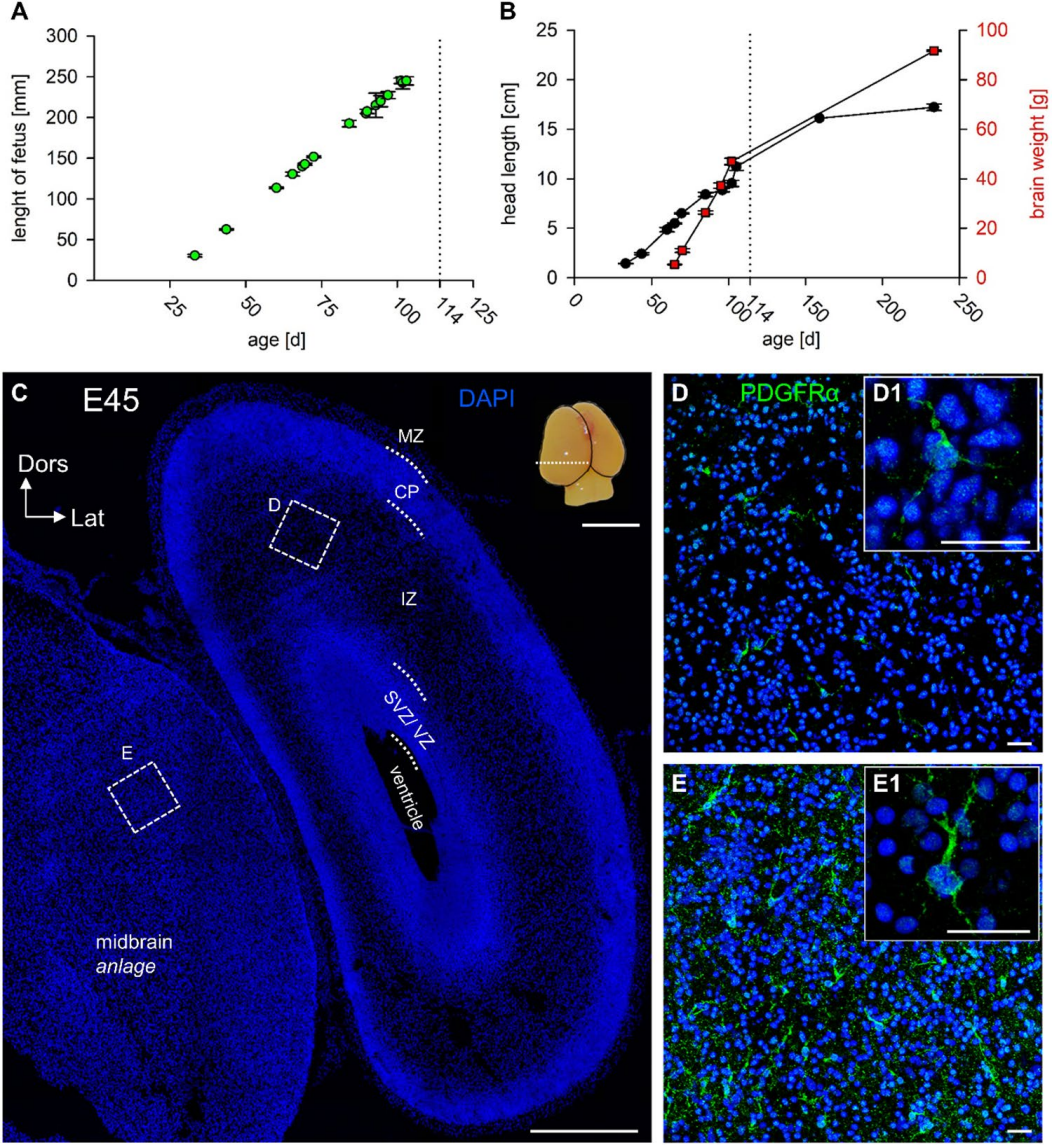
Fetal body and brain data and early oligodendrocyte precursor cells at E45. **A** Crown-rump-length (CRL) of fetuses (green symbols: Average per litters with s.e.m.) plotted against age with the formula published for European wild boar (Henry 1968). **B** Head length (black, in centimeter) and brain weight (red, in gram) plotted against fetal and postnatal age (in days). Symbols represent the average per litter with s. e. m.. **C** Tile scan of cortical section at a caudal level with midbrain *anlage*. The approximate position of the section is indicated in the E45 brain inset with a line. Dotted rectangles indicate positions of the ROIs shown in **B** and **C**. Layers are indicated. In this and the following figures: Dors, dorsal and Lat, lateral. **D** PDGFRα-ir progenitor cells in the IZ. The cell in the inset D1 has thin weakly stained processes emerging from its cell body. **E** PDGFRα-ir progenitors in the midbrain *anlage*. The cell in the inset E1 appeared more differentiated compared to the cell depicted in D1. Cell numbers in IZ and midbrain *anlage* are given in the text. Scale bars: 250 µm in **C**; 25 µm in **D**, **E**; 1 cm for the E45 brain image

Domestication has resulted in a substantial reduction of brain weight by about 41% in pig (Böndel 2017). Therefore, our model of choice is the non-domesticated form of *Sus scrofa*, the European wild boar. Staging was done using the crown-rump-length and the published formula for European wild boar supplemented by external features (Henry 1968). The litter mates had somewhat variable body lengths (Fig. 1A) which were averaged for staging the litter. For a subset of the litters, the weight of the brains including olfactory bulb and brain stem separated at the level of the foramen magnum from the spinal cord were determined. Brain weights were plotted against the head length measured in lateral view from mid-planum rostrale to the most caudal point of the head (Fig. 1B). Both measures increase steeply as expected. Of note, wild boar fetuses of the E100 litters had an average brain weight above 40 g. This is higher than the 34 g-average brain weight reported for newborn (~ E114) domestic piglets (Desantis et al. 2021). Similarly, brains of neonatal wolves are about 10% larger than brains of neonatal dogs of matching body weight, and they grow 34% larger in the adult (Schleifenbaum 1973). In Table 1, “gestation” is the proportional age with birth in pig at E114 set to 1; it has been calculated for comparison with fetal stages of other mammals.

### Tissue processing and immunostaining procedures

As described (Ernst et al. 2018) dissected cortex slabs sampled from the occipital cortex were immersion-fixed in 4% paraformaldehyde in 0.1 M phosphate buffer pH 7.4 with of 5% (vol/vol) water-saturated picric acid for about 2 weeks at 8 °C (refreshed once). After cryoprotection, tissue slabs were stored frozen in TissueTek at − 80 °C until cutting. Coronal 25 µm cryostat sections of dorsolateral parietal and occipital neocortex mounted on silanized slides were submitted to antigen retrieval (40 min citrate buffer pH 5–6 at 80 °C and cooling down slowly for about 1 h) followed by immunohistochemistry as described (Sobierajski et al. 2022). Antibodies are listed in Table 2. Immunofluorescent labeling was done after quenching the autofluorescence with Sudan Black B 0.2% (w/v), dissolved in 70% isopropyl alcohol for about 60 min before incubation of the secondary. A brief incubation with DAPI was done before coverslipping to delineate cortical layers and mark cell nuclei. Alternatively, we used biotinylated secondaries followed by avidin–biotin-horseradish peroxidase complex and 3,3-DAB. The DAB reaction product was intensified with OsO4 in phosphate buffer for 1–2 min. In some cases, a light Nissl counterstain with thionin was done followed by dehydration and coverslipping with DPX. No specific staining was seen after omitting the primary and/or secondary antibodies except for autofluorescence in blood cells.

**Table 2:** Antibodies and reagents in immunohistochemical staining

Primary antibodies	Species, label; method, lysis buffer	Source, order number, RRID	Dilution
Myelin-associated glycoprotein	Mouse hybridoma supernatant; IF	Gift of Prof. Jacqueline Trotter, University Mainz, Germany Poltorak et al. (1987), clone 513	1:10
Myelin-associated glycoprotein	Mouse; WB (RIPA)	Abcam, Cambridge, UK, Cat# ab89780, RRID: AB_2042411	1:500
Myelin basic protein	Rat hybridoma supernatant; WB (RIPA)	Gift of Prof. Jacqueline Trotter, JGU Mainz, Germany	1:700
Myelin basic protein	Rat; IF, WB (RIPA)	Bio-Rad Laboratories Inc., Feldkirchen, Germany, Cat# aa82-87, RRID: AB_325004	1:100
Proteolipid protein PLP/DM20	Mouse; IF, WB (non-SDS lysis)	Millipore, Cat.No. MAB388; RRID: AB_177623	1:1000
Olig2	Rabbit; IF, WB (RIPA)	ThermoFisher, Cat.No PA5-85734; RRID: AB_2792873	1:1000
Platelet-derived growth factor receptor alpha	Rabbit; IHC, IF, WB (RIPA)	Santa Cruz Biotechnology, Dallas, Texas, USA, Cat# sc-338, RRID: AB_631064	1:50 (IHC) 1:500 (WB)
β-actin (housekeeping protein)	Mouse; WB (RIPA, non-SDS lysis)	Sigma-Aldrich, St.Louis, MO, USA, Cat# A1978, RRID: AB_476692	1:6000
Vimentin	Mouse; WB (RIPA)	Sigma-Aldrich, St.Louis, MO, USA, Cat# V6630, RRID: AB_477627	1:1000
Glial fibrillary acidic protein	Rabbit; WB (RIPA)	Dako A/S, Glostrup, Denmark, Cat# Z0334, RRID: AB_10013382	1:1000
NeuN	Mouse; IF	Merck (Millipore), Darmstadt, Germany, Cat# MAB377, RRID: AB_2298772	1:2000
Parvalbumin	Mouse; IF	Swant AG, Burgdorf, Switzerland, Code Nr. 235	1:300
Secondary antibodies			
Anti-rabbit	Goat; biotin; IHC	Dako Hamburg, Germany, Cat# E0432, RRID: AB_2313609	1:1000
Anti-rabbit	Donkey; Alexa-488; IF	Abcam, Berlin, Germany, Cat# ab150073	1:1000
Anti-rabbit	Donkey; Alexa-488; IF	Thermo Scientific, Waltham MA, USA, RRID: AB_2687506	1:1000
Anti-rabbit	Goat; alkaline phosphatase; WB	Dako Hamburg, Germany, Cat# D0487, RRID: AB_2617144	1:2000
Anti-mouse	Goat; biotin; IHC	Dako Hamburg, Germany, Cat# E0433, RRID: AB_2687905	1:1000
Anti-mouse	Sheep; biotin; IHC	Amersham via GE Healthcare Life Sciences, Braunschweig Germany, Cat# RPN1001, RRID: AB_1062579	1:200
Anti-mouse	Goat; Alexa-568; IF	Invitrogen, Carlsbad, CA, USA, RRID: AB_2534013	1:1000
Anti-mouse	Rabbit; alkaline phosphatase; WB	Dako Hamburg, Germany, Cat# D0314	1:5000
Anti-rat	Goat; biotin; IHC	Vector Laboratories, Inc., Burlingame, CA, USA, Cat# BA-9400, RRID: AB_2336202	1:1000
Reagents			
ABC reagent	Horseradish peroxidase; IHC	Vector Laboratories, Inc., Burlingame, CA, USA, Cat# PK-6100, RRID: AB_2336827	6 µl vector A/B per mL TBS
DAPI	Fluorescent counterstain	Sigma-Aldrich, St.Louis, MO, USA, Cat# D9542	1:2500
Sudan Black	To quench autofluorescence	Merck, Darmstadt, Germany, Cat# 1387	0.2% (w/v)
DPX	To coverlip sections	Merck, Darmstadt, Germany, Cat# 06522	
Diaminobenzidine DAB	Chromogen	Sigma Aldrich, Steinheim, Germany Cat# D12384	0.02%
Osmium tetroxide	Intensification	Sigma Aldrich, Steinheim, Germany, Cat# O5500	1%

*IHC* immunohistochemistry with DAB staining, *IF* immunofluorescence, *WB* western blot

### Silver staining

As an antibody-independent method, the highly sensitive, myelin-specific Gallyas staining was employed. Sections were rehydrated 2 × 5 min with distilled water, pretreated in pyridine/acetic anhydride (2:1) for 50 min, rinsed with 50% and 25% ethanol each for 3 min, rinsed with 0.05% and 0.1% acetic acid for 3 min and with 0.5% acetic acid for 10 min. After that, sections were incubated in disposable plastic dishes with shaking in the dark for 45 min with 24 mM ammonium nitrate, 12 mM silver nitrate and 0.02% sodium hydroxide in distilled water. Incubation was stopped in 0.5% acetic acid for 10 min. Sections were developed at 22 °C for 4–5 min in 0.07% formaline, 236 mM sodium carbonate, 12 mM ammonium nitrate, 6 mM silver nitrate and 3.5 mM silicotungstic acid in distilled water. Development was stopped in 0.5% acetic acid for 2 min followed by exposure to 0.2% potassium ferricyanide for 10–15 min. The reaction product was stabilized with 0.5% sodium thiosulfate for 1 min. Sections were rinsed with 3 × 5 min with distilled water, dehydrated with ethanol and isopropanol, cleared and coverslipped with DPX.

### Western blot

Cortex at E65, E80, E95, E100 and P90 (2–3 litter mates/age) was dissected and about 5 × 5 mm blocks of apex gray matter down to the white matter were cut from SC (Craner and Ray 1991) and VC (Adrian 1943). Tissue was frozen on dry ice in Eppendorf tubes and stored at − 80 °C. Slabs containing the same regions from the opposite hemisphere were immersion-fixed and used for histology (in the present study with focus on occipital structures). For lysis, a tissue block was first crushed to near powder on dry ice. Powder aliquots were stored at − 80 °C. Microspoon portions of powdered SC and VC were lysed in RIPA buffer and gel runs (30 µg protein/lane) and immunostaining of the horizontal membrane strips harboring the protein of interest were done as described (Engelhardt et al. 2018). SC and VC samples of the selected ages were run on the same gels to compare development of the two cortical regions. Band intensities were normalized to same-lane β-actin or β-tubulin which served as control for loading equivalency. Every marker was determined twice per lysate, and normalized values were pooled to avoid technical replicates. For every marker, 2–12 independent lysates of cortex of two animals (mostly from one litter) per age were tested. Blots were photographed and relative band intensities were determined with Image J. Finally, values were expressed relative to the P90, which was considered as “adult”, and which has been set to 1.

### Analysis

DAB-stained material was analyzed with light microscopy. Plots were done from Nissl-stained sections with the Neurolucida (MicroBrightField, Inc., Williston, Vermont, USA) to indicate where brightfield photomicrographs were taken with a Zeiss Axiophot equipped with a CCD camera (PCO, Kelheim, Germany). Plots were arranged with Adobe Photoshop® and Inkscape (open-source software). Regions of interest were selected with the aim to document the distribution of immunolabeled structures in the laminar compartments. Fluorescent images and tile scans were done with a Leica TSC SP5 confocal microscope (10 × and 40 × objective with 1.1 NA, 1024 × 1024 px). Global whole-picture contrast-, brightness-, color intensity- and saturation-settings were adjusted with Adobe Photoshop®. Scale bars were generated with ImageJ (MacBiophotonics) and inserted with Adobe Photoshop® (CS6 Extended, Version 13.0 × 64). To prove colocalization of parvalbumin and MBP, yz and xz axes of confocal stack were included.

Quantification of PDFGFRα + cells and nearest neighborhood analysis was done as described for the assessment of microglia (Sobierajski et al. 2022). Regions of interest were placed over the selected cortical compartments of the confocal images. Immunofluorescent cells with a DAPI-stained nucleus in the optic plane were individually marked in the “3D-environment” function of Neurolucida 360, and calculated per mm^2^, or analyzed for the distance to their next neighbors, respectively. Data management was done in Microsoft Excel 2010. Graphs were made with SigmaPlot 12.3 (Systat Software GmbH).

## Results

### PDGFRα positive cells decline with age

PDGF is released by neurons and astrocytes to enhance oligodendrocyte progenitor cell (OPC) proliferation and inhibit premature differentiation. In rodent and human, PDGFRα is expressed very early in the lineage in progenitors and downregulated when OPCs start to differentiate into mature OLs (Noble et al. 1988; Pringle et al. 1992). At E45, the overall PDGFRα positive progenitor cell density was extremely low. Most labeled cells (on average 21.4 cells/mm^2^) were scattered in the IZ (Fig. 1C, D, D1). A few cells were seen along the lower aspect of the cell-dense cortical plate bordering the emerging subplate (on average 9.7 cells/mm^2^). MZ, SVZ and VZ were entirely void of labeling. A substantially higher cell density was already present in the adjacent midbrain *anlage* (on average 87.9 cells/mm^2^) and cells appeared somewhat more differentiated (Fig. 1C, E, E1).

**Fig. 2:**
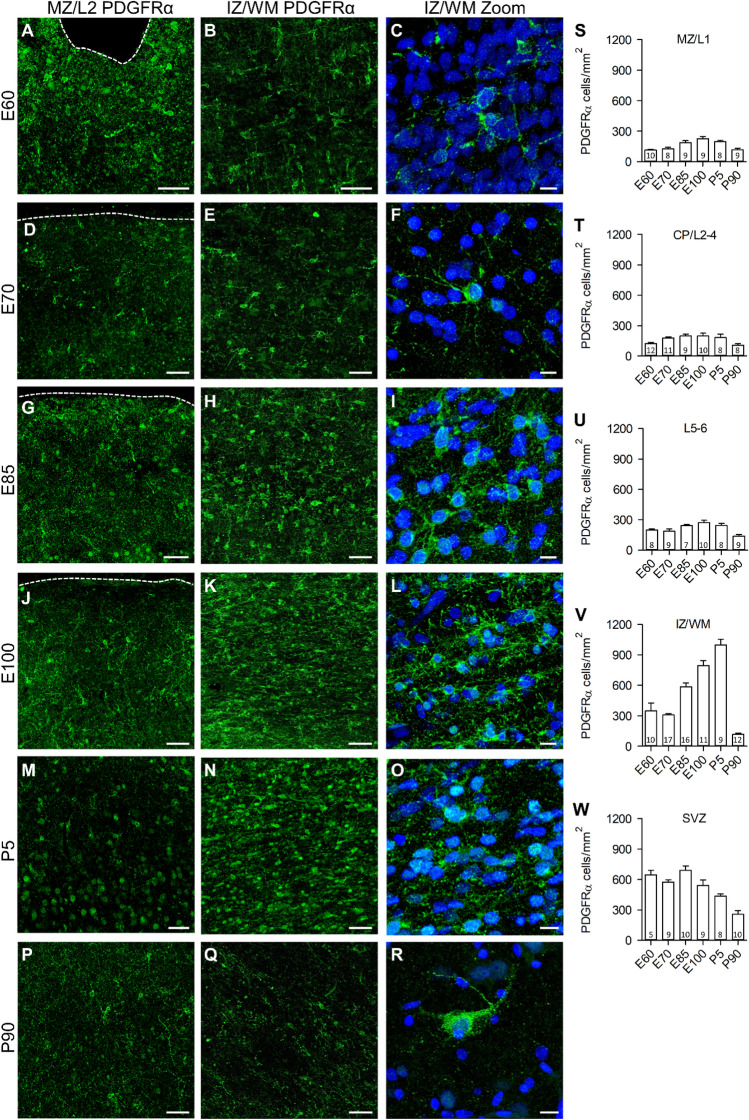
Qualitative and quantitative analysis of PDGFRα positive OPC. **A–R** Representative confocal images of marginal zone/layer 2 (MZ/L2) in the left column, intermediate zone/white matter (IZ/WM) in the middle column, and the magnification of PDGFRα positive progenitor cells of IZ/WM with additional DAPI staining in the right column. Rows: the ages E60 to P90 as indicated. Note the rather small increase and decrease in the gray matter L2/3 and L5/6 in contrast to the massive increase in the IZ/WM until E100 and P5. Dashed line marks the pial surface. **S–W** The density of PDGFRα positive progenitor cells in the laminar compartments at the selected ages as determined in 25 µm thick sections [*n* = 2–3 sections] with 5–17 regions of interest/compartment. Plotted is the number of labeled cell bodies with a DAPI-stained nucleus in the plane of the optical section per mm^2^, and represented as mean ± s. e. m.. Scale bars: 50 µm in the first two image columns, 10 µm in the third image column

A qualitative and quantitative assessment of PDGFRα positive OPC is presented in Fig. 2A-R and Fig. 2S–W, respectively, with representative photomicrographs of the MZ/CP and IZ/WM. At E60, substantial numbers of OPC populated all laminar compartments (Fig. 2A–C) indicating a massive proliferation of precursors before midgestation. Comparatively low OPC densities were counted in MZ and L2/3 (Fig. 2S, T). Somewhat higher OPC densities occurred in L5/6 (Fig. 2U). From E70 to E100 (Fig. 2D–L) the cell body density moderately increased in the gray matter followed by a decrease at P5 and P90 (Fig. 2M–R) to numbers as low or lower than those at E60 (Fig. 2S–U). Concurrent with the prenatal increase, the neuropil became progressively filled with a dense network of fine processes with punctate membrane-associated labeling. Many more OPC were present in the IZ/WM already at E60, increasing to peak numbers at P5 followed by a steep decline to P90 (Fig. 2V). An even higher OPC density occurred in the SVZ at E60, remained on a plateau until E85, and declined at E100, P5 and P90 (Fig. 2W). This suggested that each laminar compartment has its own history of oligodendrocyte occupation starting from the IZ upwards as well as downwards towards the ventricle.

### Development of myelinating cells and myelin sheaths in VC

MAG, MBP and PLP/DM20 are reliable markers for differentiating, pre-myelinating oligodendrocytes as well as mature oligodendrocytes and myelin sheaths along axons. PLP/DM20 may not be selective for the oligodendrocyte lineage, in particular during early development. It is for instance expressed in Schwann cells of the peripheral nervous system and cell body-restricted forms have been reported to occur also in neurons. At E60, neither immunopositive nor Gallyas-stained myelin structures were detected. The Gallyas method also failed at E70, possibly because the myelin was not yet compact enough to become impregnated.

**Fig. 3:**
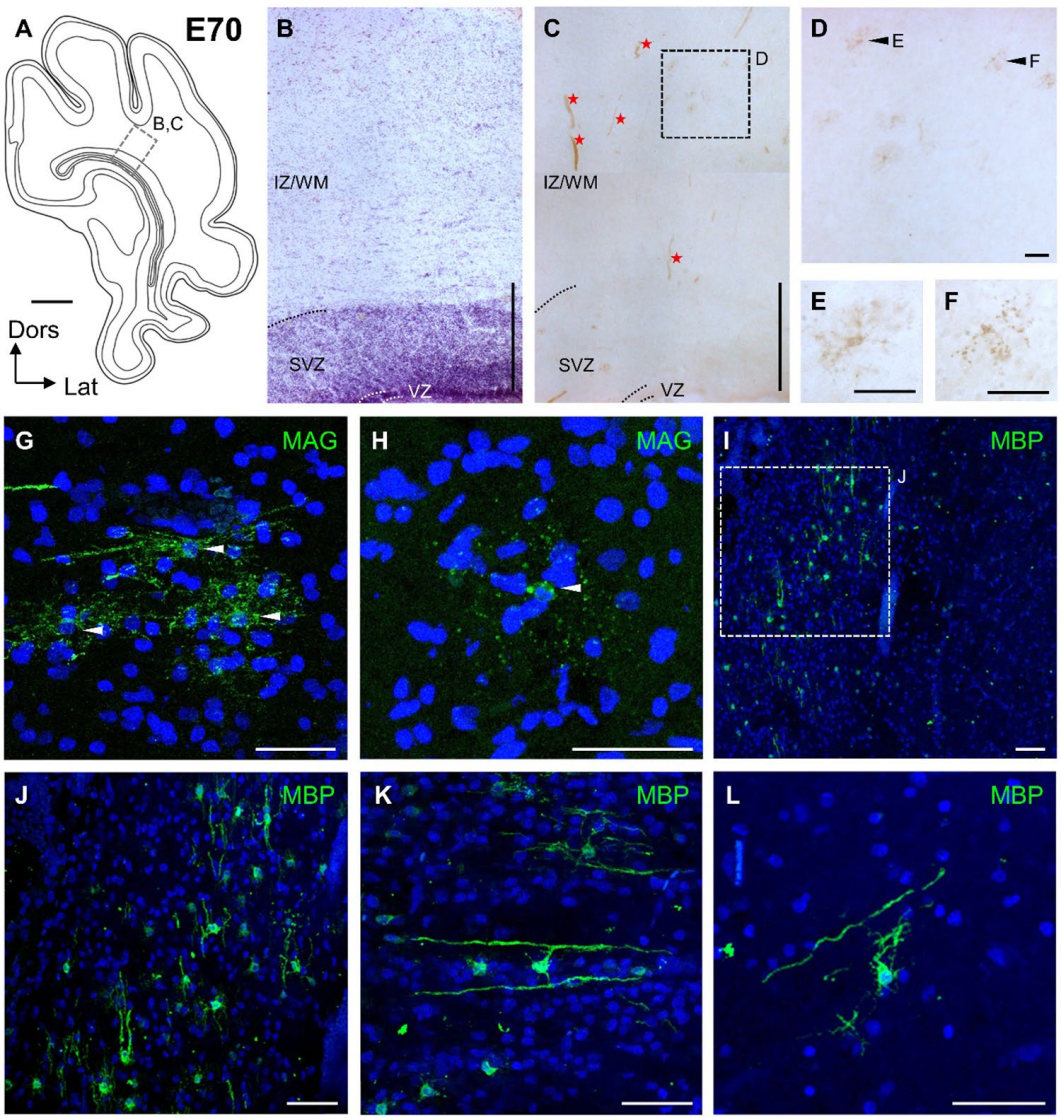
Begin of oligodendrocyte maturation at E70. **A** Schematic presentation of cortical section at the level of the visual cortex, and caudal pole of hippocampus. Dotted rectangle indicates position of the ROIs shown in B and C. **B** Thionin staining of an alternating section at a corresponding position. **C–H** MAG immunostaining. **C** Low magnification of MAG-positive elements in the IZ; DAB staining. Cells marked with arrow heads are depicted in **D**–**F**. SVZ and VZ were nearly void of immunoreaction except for unspecific labeling of blood vessels (red asterisks). **D** Weakly MAG-positive oligodendrocytes in the IZ. **E** Healthy-looking immature oligodendrocyte. **F** Oligodendrocyte with signs of degeneration. **G** Three immature oligodendrocytes with fine punctate process labeling (arrows). **H** Oligodendrocyte with signs of degeneration; arrowhead points to the cell body. Note that in this and the following photomicrographs cell bodies stain well for MBP but barely for MAG. **I–L** MBP staining in the IZ; immunofluorescence, confocal images. **I** Oligodendrocyte with long thick processes suggesting ensheathment of axons. **J** Upper IZ with few weakly positive cell bodies. **K** Enlargement of the cell shown in the box in **J**. Note its sparse processes, yet it resides next to an about 100 µm long myelin sheath. **L** Deeper IZ with a cluster of stained oligodendrocytes. Nuclei in **G**–**M** are co-stained with DAPI. Scale bars: 2000 µm in **A**; 250 µm in **B**–**D**; 50 µm in **E**–**M**

At E70, MAG and MBP antibodies yielded immunopositive cells (Fig. 3A-L). Nearly all were in the IZ/WM scattering into the SVZ. This was in line with IZ/WM and SVZ harboring the highest numbers of PDGFRα at E60 and E70. Only few cells were observed closer to the cortical gray matter, and none were detected in deep cortical layers, cortical plate, or MZ. Immunoperoxidase and immunofluorescence yielded comparable staining patterns as shown for MAG-stained oligodendrocytes (Fig. 3C–F and G–H). Most cells had faintly MAG-labeled somata having either no or a few fine punctate-stained processes, or thicker processes. The reaction product was enriched at the branch points of the processes. Some cells seemed to undergo cell death as indicated by their shrunken cell bodies surrounded by an irregular cloud of intensely stained puncta which covered the same neuropil region as the process halo of intact pre-myelinating oligodendrocytes (Fig. 3F, H). Such a nuclear condensation and disintegrating processes resemble oligodendrocyte degeneration described in postnatal rat cingulate cortex (Trapp et al. 1997). MBP staining in contrast yielded intensely stained cell bodies (Fig. 3I–L). Some oligodendrocytes had already formed sheaths parallel to the main fiber direction in IZ/WM (Fig. 3I–K) and occasionally myelin sheaths were found in the upper WM/subplate (Fig. 3L). This was suggestive of a start of myelination at 0.61 gestation.

**Fig. 4:**
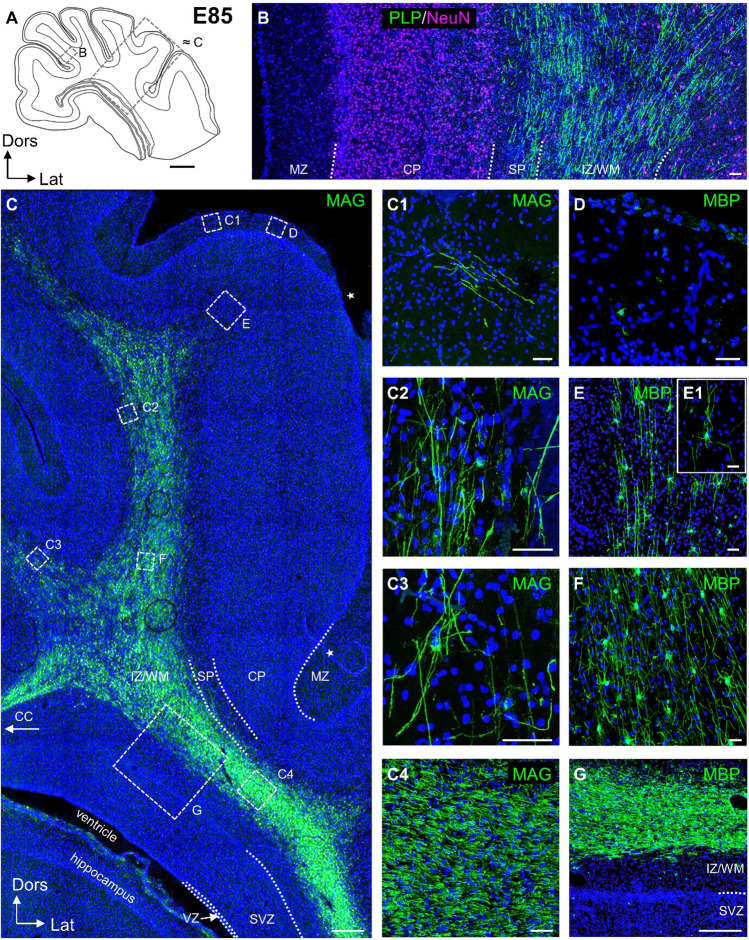
Myelination at E85. **A** Schematic presentation of cortical section at the level of the visual cortex with emerging hippocampus. Dotted rectangles indicate position of the ROI shown in **B** and **C**. **B** PLP/DM20 -ir myelin fibers of a dorsal gyral flank, photomontage of four images. Neurons are labeled with NeuN. Layers are indicated. **C** Tile scan documents the gradient of MAG staining from deep IZ/WM into gyral WM. Strongest reactivity was towards the temporal cortex (lower right) and a compact bundle extended towards the corpus callosum (CC). Subplate (SP) and gray matter are nearly void myelin. Insets **C1–C4** are taken from this section and are from MZ, infragranular layers, and upper white matter/subplate of dorsal cortex, and deep WM of the lateral cortex. They are shown at higher magnification to the right. Note in **C1**, that the first clusters of myelin are detectable in the MZ. Small artifacts (thread of overlapping pia, loss of tissue in MZ) are marked with white asterisks. **D–G** MBP immunofluorescence of an alternating section, position of ROIs is indicated in **C**. **D** Isolated cell body in the MZ. **E** Cell bodies and weaky labeled myelin sheaths in gray matter L5/6. The inset depicts a cell with radial extensions towards the pial surface.** F** Cell bodies and intensely labeled myelin sheaths in deeper gyral WM. **G** High density of cells and sheaths in IZ/WM. SVZ and VZ are largely void of staining. Nuclei are co-stained with DAPI. Scale bars: 2000 µm in **A**; 500 µm in **C**; 250 µm in **G**; 50 µm in **B**, **C1-C4 D, E, F**; 10 µm in **E1**

At E85, PLP/DM20, MAG and MBP positive myelin sheaths were observed in IZ/WM (Fig. 4A–G). A gradient of declining staining intensity and density of myelinated axons was seen from the deep white matter towards the gyral white matter (Fig. 4B, C, insets C1–C3). Cell bodies were weakly labeled with MAG and PLP, but intensely with MBP. The first cell bodies and patches of myelin were detectable in the MZ (Fig. 4C1, D), albeit at very low frequency. Myelinated axons now ran in subplate and entered L6 (Fig. 4C2, C3, E). Strongest staining was seen in compact WM of lateral cortical aspects (Fig. 4C, F). Now, also the Gallyas method detects myelin sheaths at the corresponding positions (Online Resource 1A), albeit still few and mostly weakly stained.

**Fig. 5:**
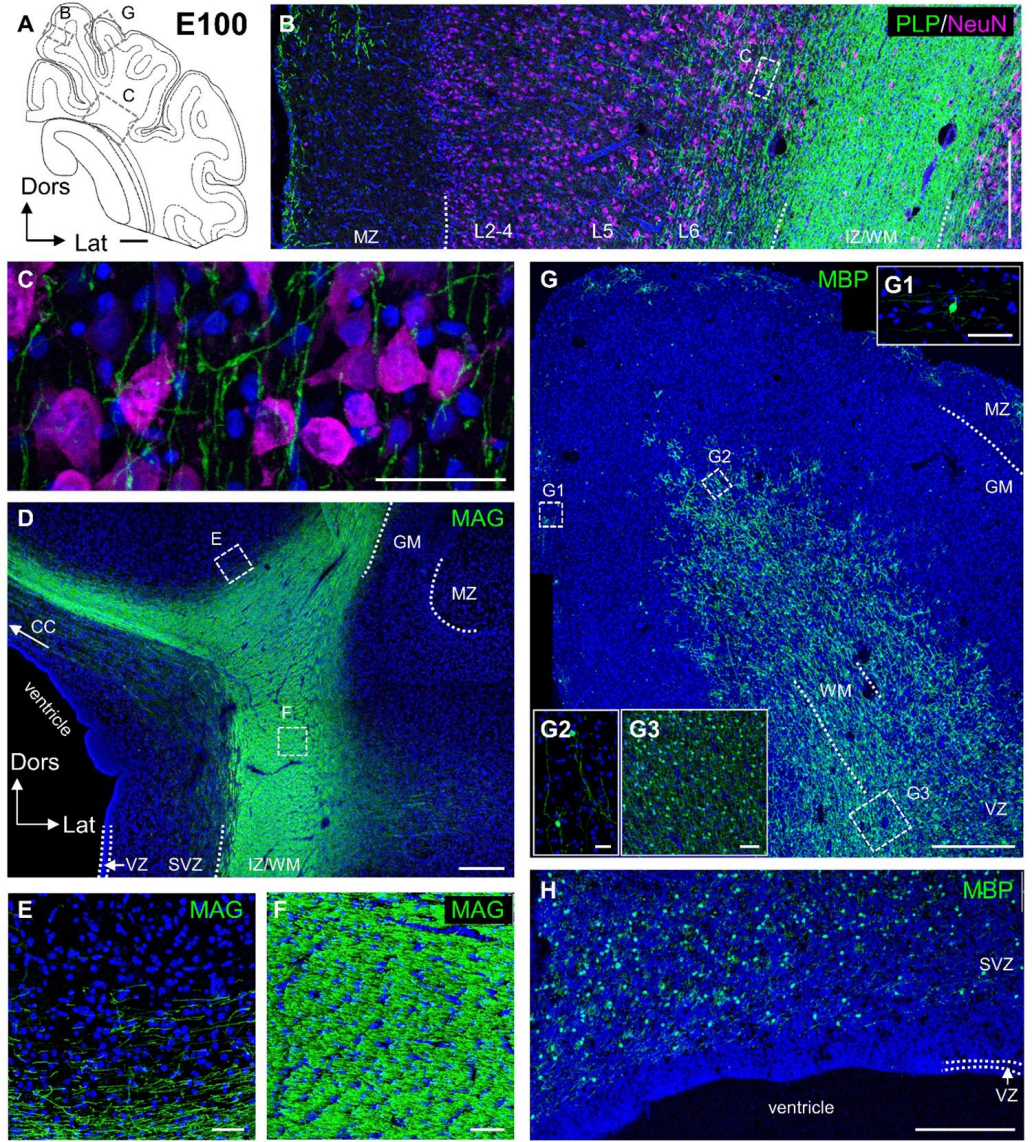
Myelination at E100. **A** Schematic presentation of cortical section at the level of the visual cortex with hippocampus. Dotted rectangles indicate position of the ROIs shown in **B**, **C** and **D**. **B, C** PLP/DM20 and NeuN immunofluorescence. **B** PLP/DM20 positive myelin fibers at dorsal cortical gyral flank, photomontage of four images. Neurons are labeled with NeuN. Layers are indicated**. C** Higher magnification of L5/6 neurons and ascending myelinated axons. **D–F** MAG immunofluorescence. **D** Tile scan to document the gradient towards the dorsal cortical gyri. Strongest reactivity was towards the temporal cortex (middle right) and a compact bundle of myelin extended towards the corpus callosum (CC). Dotted lines show borders of cortical layers. **E** Higher magnification of upper WM at a gyral flank. **F** Higher magnification of deep WM of the lateral cortex. **G, H** MBP immunofluorescence. **G** MBP at apex of gyrus, photomontage of six images. Insets **G1**–**G3** are from MZ, infragranular layers and upper WM. **H** SVZ occupied by labeled cell bodies and processes. VZ was void of staining. Nuclei are co-stained with DAPI. Scale bars: 2000 µm in **A**; 500 µm in D; 250 µm in **B**, **G**, **H**; 50 µm in **C**, **E**, **F**, **G1–G3**

At E100, the density of myelinated axons had substantially increased (Fig. 5A-H). Immunofluorescence revealed compact myelin in the lateral deep white matter and towards the corpus callosum. The white matter of all gyri now contained a high density of MBP-stained cell bodies beginning to arrange in rows parallel to the main fiber direction. Myelin sheaths filled the WM and extended well into infragranular layers intermingling with NeuN-labeled neurons (Fig. 5B–E, Online Resource 1B). Supragranular layers were still largely void of myelin sheaths and harbored only few scattered pre-myelinating oligodendrocytes. Degenerating cell bodies were rarely seen in WM, which could be due to masking by the compact myelin (Fig. 5D, F). A few degenerating cells occurred in GM along the ascending front of myelination, in accordance with reports in gray matter of P11 rat cortex (Trapp et al. 1997), and with a spatial distribution matching our stainings at E100 in pig cortex. Cell bodies with horizontally oriented processes as well as horizontally oriented myelin sheaths had increased in the MZ (Fig. 5G1). Their density varied from gyrus to gyrus presumably reflecting the connectional maturation of the underlying cortical areas. MBP positive oligodendrocytes and myelin sheaths began to occupy the SVZ (Fig. 5H) suggestive of gradual replacement of the SVZ by WM. The VZ remained void of labeled cells bodies and sheaths. The general pattern of myelination in pig cortex two weeks before birth reflects the adult pattern and corresponds to the pattern reported for rodents well after the second postnatal week.

**Fig. 6:**
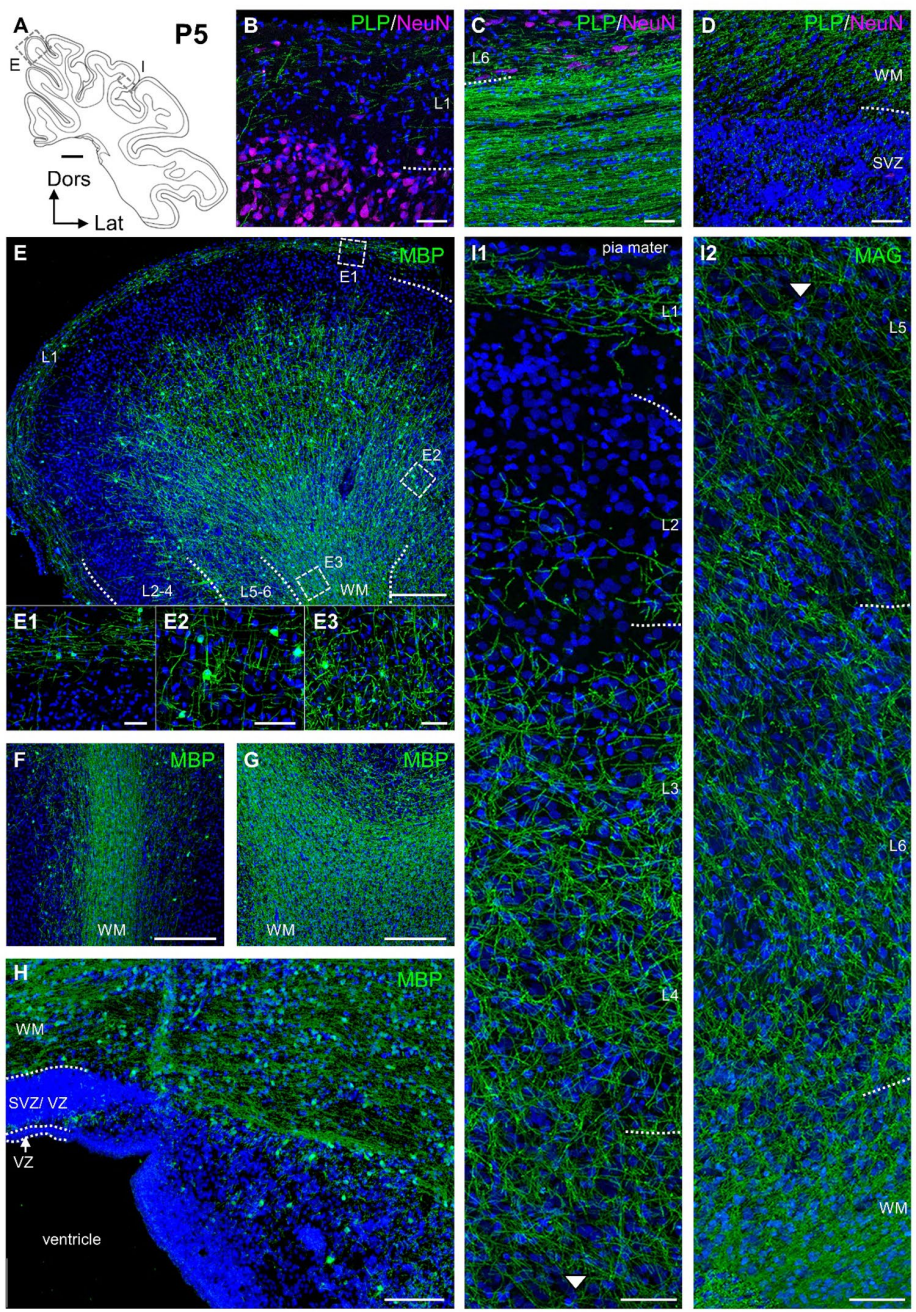
Myelination at P5. **A** Schematic presentation of cortical section. Dotted rectangles show positions of ROIs shown in E and I. **B–D** PLP/DM20 and NeuN- immunofluorescence. **B** MZ and L2. **C.** WM at border to L6. **D** WM and SVZ. **E–H** MBP immunofluorescence. **E** Gyrus, photomontage of six images. Insets **E1**–**E3** are from MZ, L5 and WM, respectively. **F** WM at flank of gyrus. **G** WM at bottom of sulcus. **H** WM, SVZ, VZ and ventricle. **I1–2** MAG immunofluorescence through the cortical depth. Nuclei are co-stained with DAPI. Scale bars: 2000 µm in **A**; 250 µm in **E**–**G**; 50 µm in **B**–**E1-3, H, I1-2**

Perinatally (a P5 domestic German Landrace piglet), myelination extended from the SVZ to the MZ as revealed by immunofluorescence and silver staining (Fig. 6A–I, Online Resource 1C). Myelinated fibers ascending from white into gray matter fasciculated as vertical bundles still largely sparing L2 (Fig. 6B, E, I1, I2). However, the gap between the ascending front of myelination and the MZ with its horizontally oriented myelin sheaths became narrower. Strongly MBP positive cell bodies now demarked the ascending front, whereas cell bodies in the WM became less well detectable presumably because the MBP immunoreactivity of the myelin sheaths dominates the antibody binding by its sheer amount (Fig. 6C, E–G). Degenerating oligodendrocytes were no longer detectable in the dense mesh of labeled structures. Oligodendrocytes and myelination spared the VZ but occupied the SVZ (Fig. 6D, H). The myelin staining pattern remained comparable to the pattern seen at E100.

Intensely MBP expressing cell bodies at E85, E100, and P5 allowed a quantitative assessment with a nearest neighbor analysis as a proxy for cell density. The average distance in the gray matter narrowed from 88.4 ± 9.3 µm to 69.5 ± 1.8 µm, and to 61.2 ± 1.3 µm at the three stages. The average distance in the IZ/WM remained fairly constant (39.6 ± 0.8 µm, 35.3 ± 0.5 µm, and 43.9 ± 1.2 µm at the three stages). Similarly, the average distance in SVZ at E100 and P5 remained constant 34.9 ± 0.9 µm and 39.6 ± 1.5 µm at the two stages (no labeling of SVZ at E85). At the older ages P30 and P90 labeled somata were no longer detectable at sufficient numbers. With respect to the enormous increase in brain volume and thus volume of the WM, this suggested that substantial numbers of oligodendrocytes become added continuously to WM and the other laminar compartments. The SVZ was void of labeling at E85, but MBP positive cells had invaded at E100, and somata continued to reside close to each other at P5. This suggested that the gradual assimilation of the SVZ into the WM begins weeks before birth.

**Fig. 7:**
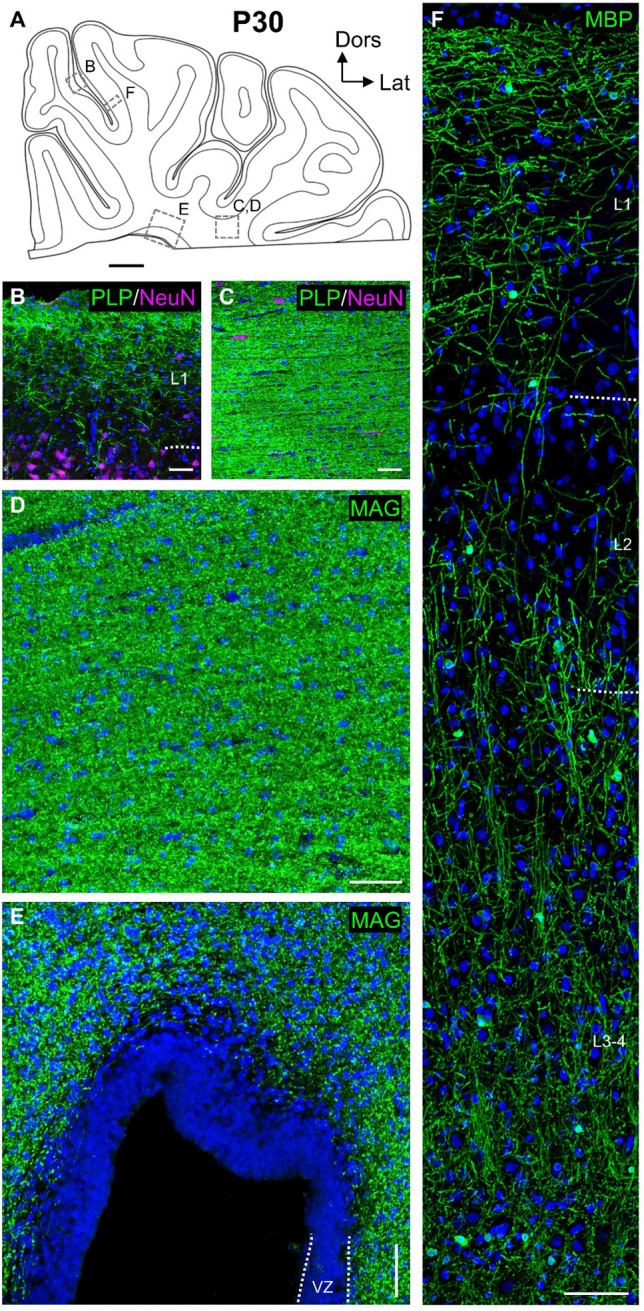
Myelination at P30 and P90. **A** Schematic presentation of a cortical section at P30 at the level of the anterior visual cortex rostral to the hippocampus. Dotted rectangles indicate position of the ROIs shown in **B**–**F**. **B** PLP/DM20 immunofluorescent myelin fibers in L1 (**B**). Neurons are labeled with NeuN. **C** PLP/DM20 immunofluorescent myelin sheaths in WM. **D** MAG immunofluorescence of compact myelin in deep WM at P90. **E** MAG immunofluorescence of fibers invading the remnants of the SVZ at P90, it transforms into WM. VZ remained void of staining. **F** MBP immunofluorescence of upper cortical layers at P90. L1 is now completely covered by myelinated axons. A narrow gap is in L2. Nuclei are co-stained with DAPI. Scale bars: 2000 µm in **A**; 50 µm **B**–**F**

At P30, the cortex had expanded even more. The myelin pattern seen with immunofluorescence and silver staining matched the pattern present at P5 (Fig. 7A–F, Online Resource 1D). Labeling intensities had increased in particular in deeper white matter and, with the silver staining, also in gray matter suggestive of more massive myelin sheaths (Fig. 7B–D). Myelination in L1 had increased (Fig. 7B, F). Sparse myelination was now present in L2 (Fig. 7F), this is generally seen in mammalian cortex. MBP still labeled somata in gray matter, but the intense labeling of the sheaths masked any weakly stained somata within the highly compacted axonal tracts. Further, myelin sheaths continued to occupy the remains of the SVZ but spared the remains of the VZ (Fig. 7E).

### Prenatal myelination of interneuronal axons

**Fig. 8:**
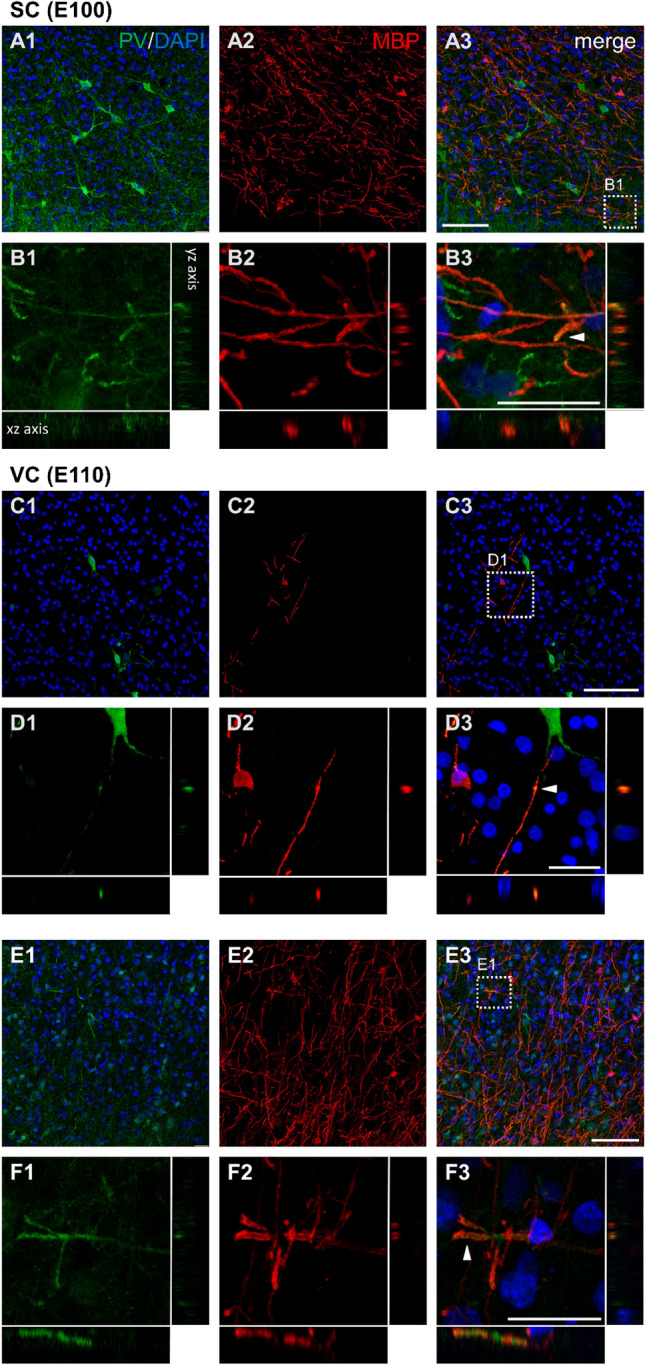
Prenatal myelination of PV positive axons in SC and VC. **A1–A3** L5 of SC at E100 densely populated with parvalbumin (green) positive somata and neurites and MBP positive fibers (red). **B1–B3** Magnification of the box in A3. Some PV axon fragments are ensheathed by MBP positive myelin shafts (arrowhead), other neurites and somata were only PV positive. **C1–C3** L2/3 of VC at E110 sparsely populated with PV cells and axons and myelin. **D1–D3** Magnification of the box in C3 shows a myelinated PV positive axon which was not arising from the adjacent PV neuron. **E1–E3** In L5 of VC at E110 somewhat more myelinated axons were found. **F1–F3** A fragment of a horizontal PV positive axon was MBP positive. Note, that the myelination is not continuous. Single channels shown in photomicrograph 1 and 2 and merged in photomicrograph 3. The xz and yz axis at 90-degree angles confirm the overlap. Nuclei are co-stained with DAPI. Scale bars: 100 µm for **A**, **C** and **E**; 25 µm for **B**, **D**, and **F**

Myelin sheaths insulate primarily afferent and efferent axons. Besides pyramidal projection neurons, however, axons of inhibitory parvalbumin (PV) positive basket cells are partially myelinated. In rodent, this myelination occurs postnatally (Micheva et al. 2021). PV expressing interneurons are present at GW 36 in the human cortex (Honig et al. 1996). We suspected that PV expression in pig cortex is also starting prenatally. We were curious to see if myelination of PV axons could be detected before birth. Looking at somatosensory cortex at E100 with co-staining of PV and MBP revealed numerous PV neurons (Fig. 8A1–A3) and scattered double-positive elements of up to 50 µm in length in L2/3 and L5. The element depicted resembles a horizontal axon branching into myelinated collaterals (Fig. 8B1–B3).

In VC at E110, the number of PV neurons was much lower (Fig. 8C1–C3) in L2/3 as well as in L5. Myelin sheaths were present and ensheathing PV cell axons (Fig. 8D1–D3; note, that the axon did not arise from the soma shown). Another example from L5 is given in Fig. 8E1–E3, and at higher magnification in Fig. 8F1–F3. It suggested that basket interneurons start to become myelinated already weeks before, and the ontogenetic delay in myelination of VC to SC accounts for axons of excitatory cells as well as axons of inhibitory neurons.

### Development of myelin protein expression in somatosensory cortex develops earlier than in visual cortex

Myelin morphology has been reported to be not affected by postmortem time, but biochemically, myelin basic protein may deteriorate due to autolysis, albeit cooling the brain reduces the degradation substantially (Fishman et al. 1977). Our fetuses had been stored and transported ice-cold until preparation. To rule out that a longer postmortem interval might negatively affect the MBP level, we sampled cortex of two neonatal rats used for slice culture preparations (Engelhardt et al. 2018), and two 14- and 16-months old rats from the in-house breeding facility. One animal each was immediately prepared, and cortex was frozen. The second animal was kept on ice for 24 h before preparing the cortex. Blots (30 µg/protein/lane) revealed no detectable MBP bands in the newborn and strong bands in the adult, as expected. Qualitatively, there was no difference between the freshly prepared and the ice-cold 24 h samples (Online Resource 2 A). Further, long postmortem storage did not affect the expression of GFAP and vimentin (Online Resource 2B, C).

**Fig. 9:**
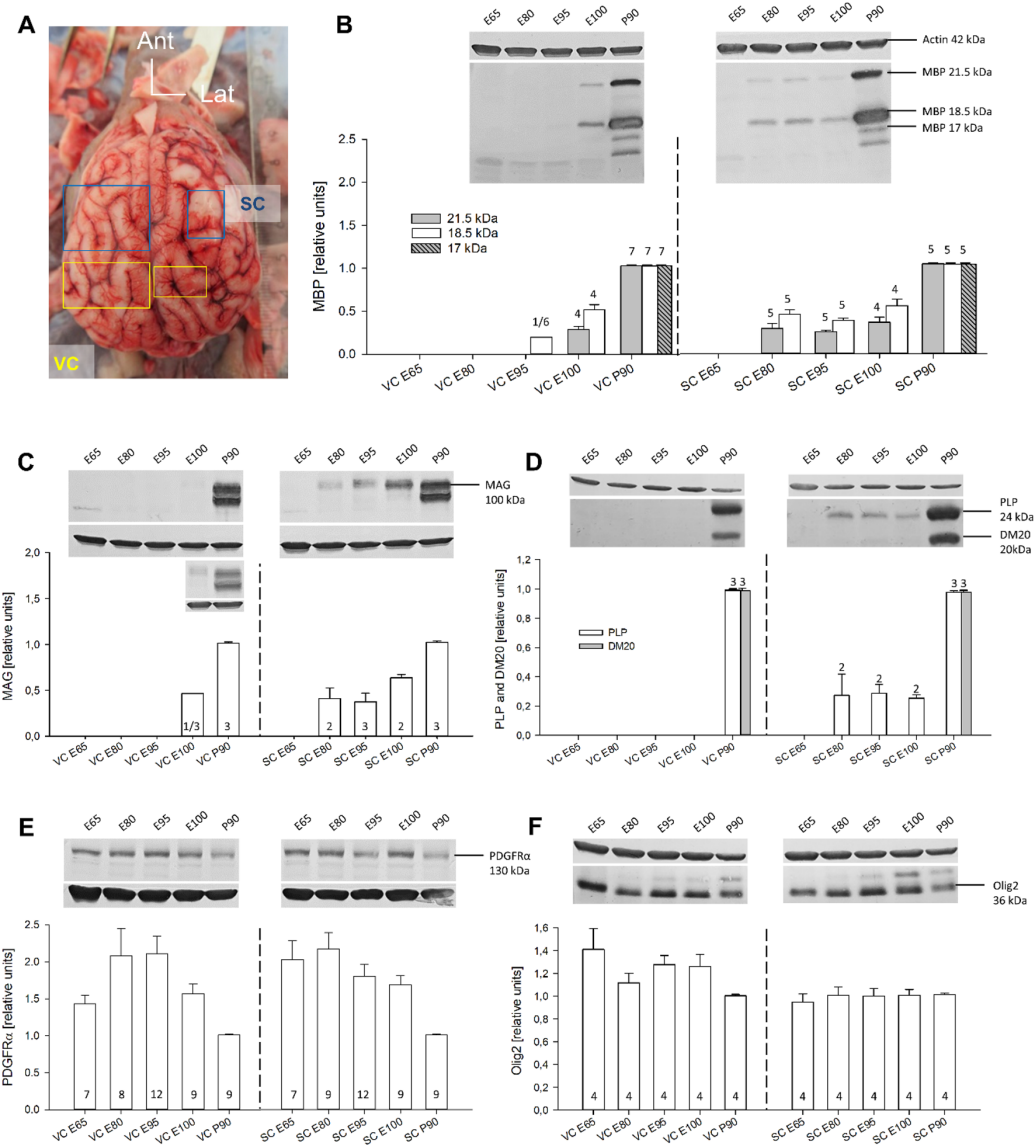
Western blot analysis of myelin proteins. **A** Developmental protein expression is shown together with representative blots (SDS PAGE). The ages analyzed were E65, E80, E95, E100, and P90. Visual (VC; left) and somatosensory (SC; right) cortex are shown side-by-side to document the area-specific expression profiles. The amount of protein in each case was normalized to the amount of the housekeeping protein actin. The protein levels were set relative to the amount in P90, which was considered as young adult. A freshly prepared E85 brain, the snout is to the top. Small rectangles demarcate visual (yellow) and somatosensory cortex (blue) gyri from which small blocks were cut down to the gyral apex white matter and frozen on dry ice. The larger rectangles demarcate the slabs for immersion fixation. **B** Expression of MBP protein. Plotted are the isoforms running at 21.5 kDa, 18.5 kDa and 17 kDa. Of VC E95, only one out of 6 lysates delivered a measurable band. **C.** Expression of MAG protein. Plotted was the 100 kDa isoform; the 70 kDa isoform could only be seen at P90. Of VC E100, only one out of 3 lysates delivered a measurable band, it is shown in the small inset. **D** Expression of PLP/DM20. A band at the predicted size of 24 kDa was detectable in SC from E80 onwards, but levels at P90 were substantially higher. DM20 at 20 kDa was only detectable in the P90 lysates of VC and SC. **E** Expression of PDGFRα protein declined with fetal age in both areas. **F** Expression of Olig2 tended to decline in VC, and was already at the lower level P90 level) in SC. The numbers in or above the bars are the number of lysates. In **B** and **C**, blots of the youngest stage revealed detectable bands only in one of the tested lysates

Human fetuses show spontaneous movements and somatosensory responses from gestational week 8 onwards which become transmitted to the cerebral cortex around gestational week 25 with the maturation of the thalamocortical projection. This input, elicited either by spontaneous muscle activity or upon intrauterine stimulation, is considered important for the formation and consolidation of the body representation (Khazipov and Milh 2018; Tau and Petersen 2010). To test the view that the somatosensory system develops early we quantified protein expression in samples from visual cortex (VC) caudal to the ansate sulcus (Adrian 1943) and from somatosensory cortex (SC) coronal gyrus overlapping the representation of the pig’s rostrum (Craner and Ray 1991) as shown in Fig. 9A. Indeed, measurable amounts of the 21.5 kDa and 18.5 kDa MBP isoforms were detectable from E80 onwards in SC. One of three tested VC lysates already reported a measurable 18.5 kDa MBP band at E95. Both isoforms were present in VC at E100. The amount is less than half of the amount present at P90. The 17.5 kDa MBP isoform was only detected in the P90 lysates (Fig. 9B).

Similarly, the 100 kDa MAG protein was detected in SC from E80 onwards whereas only one of three tested VC lysates displayed a measurable MAG band at E100 (Fig. 9C). As reported (Pedraza et al. 1991; Lai et al. 1987), the larger MAG isoform occurred first, and a smaller isoform was present at P90.

The PLP/DM20 protein was detectable at 24 kDa in SC from E80 onwards (Fig. 9D) and the amounts were low compared to P90. The DM20 variant of PLP was only detectable at P90. In VC, the blots detected the two bands only at P90. DM20 is a splice variant occurring at lower levels but coregulated with PLP (Nave et al. 1987). DM20 is more enriched in ventral CNS and peripheral nervous system which may explain the failure to detect above-threshold amounts in cortical lysates.

The comparison of the two areas comes with a certain limitation due to the sampling strategy. We sampled the apex region (gray and apex white matter of gyri in SC and VC. Therefore, with the protein blots, we quantify the time course of myelination of gray matter and apex white matter. We assumed that this reflects areal maturity better than an expression profile including deep fiber tracts belonging to unidentified regions. The beginning of cortical myelination in deep white matter and IZ/SVZ is represented qualitatively by the immunostaining, and indeed, earliest MAG-positive cells were seen in the IZ already at E70.

Expression of PDGFRα increased in VC from E65 to E80 and started to decline at E100. In SC, levels were high at E65 and E80 and started to decline already at E95 (Fig. 9E). The curve in VC was shifted to older fetal ages. The decline matched the cell counts (Fig. 2), yet PDGFRα cells persisted at P90 and the protein remained well detectable. In rat cortex, PDGFRα expression declines after P7 (He et al. 2009). Olig2 expression tended to be higher in VC at E60 and declined to the lower level seen in SC from E60 onwards through P90 (Fig. 8F). Oligodendrocyte progenitors are known to remain throughout life. Taken together, progenitors, myelinating cells and myelin protein expression were observed earlier in somatosensory than in visual cortex. For PDGFRα and Olig2, the limitation was the lack of fresh brain material younger than E65. Therefore, we missed expression onset in the blot analysis. However, the immunostaining revealed the first PDGFRα positive cells in IZ and lower CP/subplate at E45 (Fig. 1), and similarly, nuclear labeling for Olig2 was detectable from E45 onwards (not shown). Such low levels of protein expression might have been barely detectable with protein blots.

As controls, we analyzed in the same lysates the expression of GFAP and vimentin, which are markers for radial glia, astrocytes, and non-neural cells (Online Resource 2D, E). Expression of 50 kDa GFAP was rather weak at E65. Thereafter, GFAP increased in VC and SC with a similar profile towards P90 (Online Resource 2D) suggesting the appearance of astrocytes. This is comparable to studies in human (Honig et al. 1996) reporting weak GFAP staining at gestational week 14 followed by a strong increase of GFAP after midgestation. The expression of 58 kDa vimentin was high already at E65 and remained at that level through P90 (Online Resource 2E). Vimentin is present very early in human fetal brain (González-Arnay et al. 2017) and stains radial processes spanning the entire cortical wall. It continues to be present in remains of the ventricular zone, in ependyma and blood vessels during the postnatal period. For occipital cortex, the glial marker expression in human resembles that of rodent (Honig et al. 1996), and pig does not seem to deviate.

### Thickness of myelin sheaths in SC and VC

**Fig. 10:**
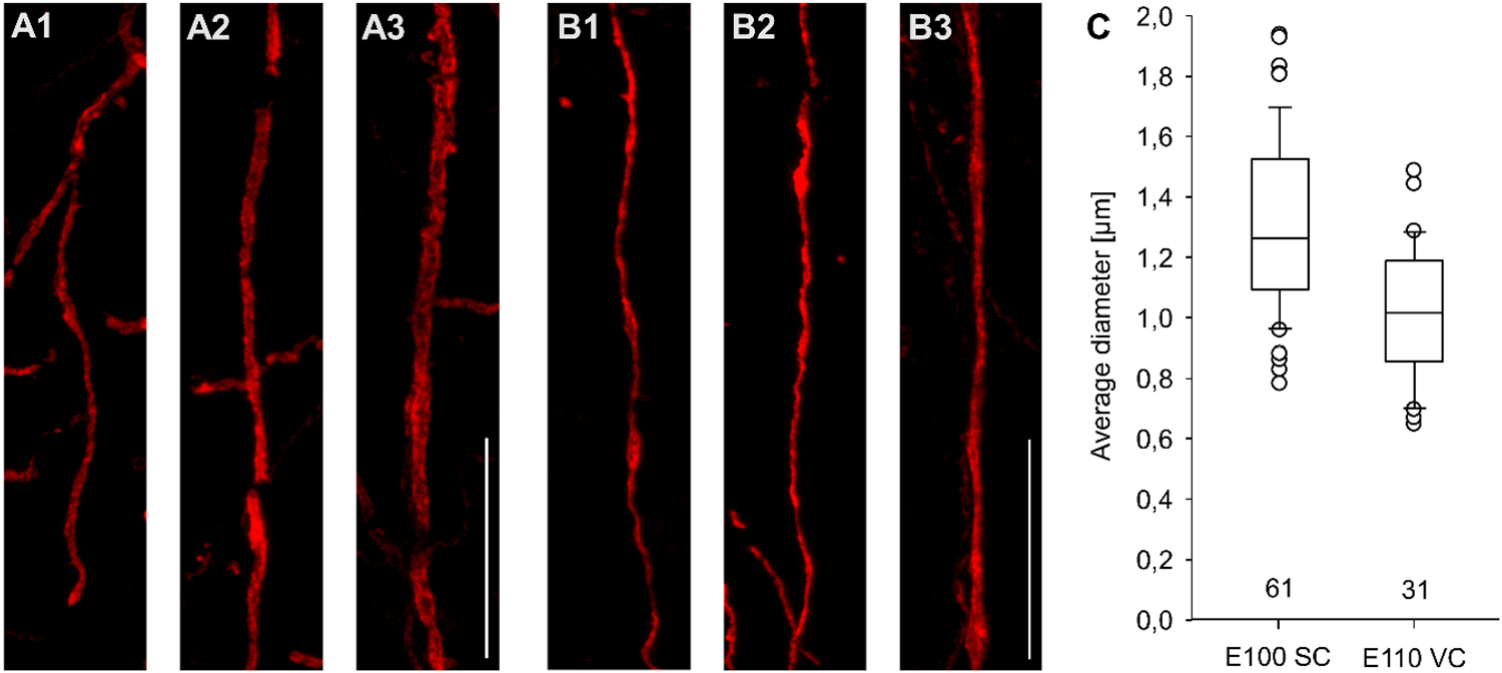
Thickness of myelin sheaths in SC and VC. **A1–A3** Myelin sheaths in SC at E100. **B1–B3** Myelin sheaths in VC at E110. The examples shown in A and B, represent rather thin (1), average (2) and large diameter sheaths (3) sampled in a vertical panel from L2 to L6. **C** Box plot showing the average diameter of myelin sheaths; every dot is one myelin sheath. The number of sheaths analyzed is given below the boxes. Scale bars: 25 µm for A and B

Finally, we wondered if the time-dependent difference observed with the protein blots is also detectable at the anatomical level. Vertically oriented myelin sheaths of minimum 50 µm length were sampled from confocal sections in vertical panels from L2 to L6, and 3D-reconstructed (Fig. 10A, B). The average diameter of every sheath was determined with the Neurolucida 360 Suite. Sheaths from E100 SC were on average 1.31 µm in diameter. Despite being already ten days older, sheaths from E110 VC were on average only 1.02 µm in diameter (Fig. 10C). This supported our observations that myelination of axons occurs earlier in SC than in VC.

## Discussion

### Species comparison

In precocial species such as the pig, cattle, sheep and guinea pig, myelin is already detectable in the prenatal brain. In domestic pig, elevated cerebroside levels suggest the presence of myelin in subcortical structures between E70-E80, with a first peak in the cortex shortly before birth, and a second peak at P30 (Sweasey et al. 1976). During this time, myelination seems to be influenced by the presence of a gut microbiome. In germ-free piglets the cortical white matter volume is reduced, density of oligodendrocytes is regionally reduced due to lower proliferation, however, protein expression of MAG, MOG and MBP is at levels seen in wildtype piglets (Ahmed et al. 2021). The steady increase of cholesterol also suggests the presence of myelin from E100 onwards in pig (Pond et al. 2000). At six postnatal months, myelination is established in prefrontal cortex. In cattle, myelin detected with immunohistochemistry for MBP, MAG, proteolipid protein, and Luxol Fast Blue staining is in cortex and corpus callosum during the sixth fetal month (about 0.65 gestation), and by the seventh month in frontal and occipital regions as well as within the gray matter (Urban et al. 1997). Sheep display myelin signals in the cerebral white matter by around E120 (about 0.83 gestation) (Barlow 1969). In guinea pig, a precocial rodent, intensely stained MBP and MAG-positive fibers are present at E60 (0.90 gestation) in the white matter and ascending to L2/3, as well as in the marginal zone (Tolcos et al. 2011). Such a pattern mirrors that of rat at 1 week postnatal and older.

In rodent, earliest PDGFRα expressing cells appear at E16 and increase in density between E18 and P10, in particular in the white matter. Proliferation continues well through the third postnatal week in rodent cortex (Moroni et al. 2018). Although cell numbers and protein decline, PDGFRα expressing cells remain well detectable in 18 months old cortex (Pringle et al. 1992; He et al. 2009). MAG is detectable in the cytoplasm of oligodendrocytes of the anterior commissure at P5-7, MBP expression and MAG-positive myelin sheaths occur during the second postnatal week (Gil et al. 2010).

In human, single cell transcriptomics detects pre-oligodendrocyte precursors in forebrain ganglionic eminences by the 8th week (~ 0.2 gestation) (Van Bruggen et al. 2022). PDGFRα immunopositive precursors are present at the 9–10th week (Jakovcevski 2009). They produce astrocytes and oligodendrocytes and migrate to the neocortex. Additional progenitors are generated by the cortical outer radial glia. In human cortical SVZ at 15th week (~ 0.37 gestation), PDGFRα marks 8–9% of all bisbenzimide-counterstained cells (Jakovcevski et al. 2009). At 20–24th week (0.5–0.6 gestation) these precursors and their progeny have populated the cortex (Huang et al. 2020). The population is considered to be equivalent to the second wave of oligodendrocytes reported for the fetal mouse (Kessaris et al. 2006). In cortex, MBP expression is seen from the 35th week (~ 0.87 gestation) onwards in the central gyrus and by the 37th week in the optic radiation (Hasegawa et al. 1992). PLP expression is earlier observable and visible in the central gyrus at the 33rd week (Iai et al. 1997). Until term the amount of myelinated white matter increases to about 5% (Hüppi et al. 1998). Myelin development in white and gray matter proceeds beyond late adolescence and, compared to postnatal chimpanzee, humans are born with lower numbers of myelinated cortical axons (Miller et al. 2012; Deoni et al. 2015).

### Within and across species differences

Staining results and reported cell densities are difficult to compare between studies. Technical differences may be one reason. In addition, the developmental status of the individual fetuses and the scarcity of fetal material for research per se may be an under-recognized aspect. Guinea pig is a model for human birth complications due to fetal hypoxia, placental insufficiency and intrauterine growth restriction. Individual body weights of growth-restricted animals may be substantially lower compared to normally developing animals. However, brain size and cortical volume were largely unaffected after growth restriction (Tolcos et al. 2011). Similar in domestic pig, overall brain development seems less affected in small-bodied versus large-bodied piglets, despite substantial differences in body organ weight (Widdowson 1971; Ashworth et al. 2001; Vallet and Freking 2006). It seems as if the brain takes what it needs at the disadvantage of the other organs. This said, in near-term fetal guinea pig with placental insufficiency, cortical myelination was strongly delayed due to a delayed maturation of oligodendrocytes; however, the deficit quickly normalizes during the week after birth (Tolcos et al. 2011). In domestic pig, within-litter weight variability has been reported for myelination between E92 and E110 such that the smallest individuals have a smaller cerebellum and brainstem as well as a lower expression of MBP mRNA and protein when compared to the largest sibling (Vallet and Miles 2012). Such deficits may contribute to piglet survival due to subtle impairments of movement or sensory processing. In line with that, advanced myelination of guinea pig fetal brainstem is correlated with an enhanced auditory brainstem response (Wang et al. 1993).

Within-litter body weight in our fetal wild boar material collection varies by a factor of 2 at the extreme (Ernst et al. 2018). We paid attention to select average-sized fetuses for the experiments to reduce potential fluctuations in expression strength. However, we can not completely rule out that some variability may be due to subtle developmental advances or delays. This may explain the finding of a measurable MAG band in only one of the three tested lysates of VC gyral apex gray matter already at E100. Also, for instance in postnatal human and chimpanzee cortex, individual differences of myelin fiber length have been reported with the highest length variability between 5 and 10 years of age (Miller et al. 2012). Although barely addressed, comparable limitations could affect rodent data as well because the time course of thalamocortical fiber maturation differs as a function of body weight, litter size and length of gestation (Hoerder-Suabedissen et al. 2008).

### Myelination and sensory plasticity

We observed myelin proteins increasing much earlier in the somatosensory than in visual cortical gray matter. This is in line with the view that sensorimotor system development begins early and is driven by activity triggered by fetal movements (Tau and Petersen 2010). Accordingly, we suggest that fetal pig cortex generates sufficient electrical activity to drive activity-dependent myelin sheath formation. The observation of more PV positive neurons and more myelinated PV positive axons in the SC than in the VC further confirmed the early maturity of the SC. Moreover, the myelin sheaths were thicker in the SC than in the VC, The partial myelination is an indicator of mature basket cells, and it is essential for fast inhibition and synchronization of cortical oscillations (Dubey et al. 2022). Neuronal activity is essential for production, secretion, and action of BDNF produced which promotes the expression of MBP (Djalali et al. 2005) as well as of PV which also depends on neurotrophins (Patz et al. 2004). Further, astrocytes recruited by ATP released from electrically active axons promote myelination (Ishibashi et al. 2006), and the substantial increase of GFAP expression from E80 onwards in VC and SC argues for the presence of astrocytes in fetal pig cortex. A recent report in mice showed that somatosensory input at late fetal stages co-activates the visual cortex, and the emerging retinal input is important to segregate sensory modalities (Guillamón-Vivancos et al. 2022). It remains to be shown if this occurs in all mammals at comparable stages, for instance explaining the take-over of visual cortex by touch (e.g. Braille reading) in blind humans. In case it does occur prenatally in pig, however, it is interesting to note that the somatosensory influence is not strong enough to synchronize protein expression between the two areas.

Myelination has been linked to ocular dominance plasticity in rodent because MBP is one of the top genes upregulated towards the end of the critical period. Our results show that VC already displays myelinating oligodendrocytes and MBP in gyral white and deep gray matter in prenatal pig lacking any visual experience. MAG, an inhibitor of axonal outgrowth and ligand to the Nogo receptors (McGee et al. 2005; Jitsuki et al. 2016) started to be expressed well before birth in SC but was barely present at E100 in VC gray matter. After eye opening in rodent and presumably after birth with open eyes in pig, visual experience serves to consolidate the functional cortical architecture which became laid out by mechanisms that do not require active vision. Therefore, myelin per se is not ending the critical period, and myelin expression, and peak and end of the critical period may simply be coincident rather than causally related. Recent data suggest that MBP regulation may not even reflect synaptic plasticity in general. In mouse SC, MBP is regulated by environmental enrichment even weeks after birth with enrichment promoting the integration of newly myelinating oligodendrocytes, whereas in VC MBP is regulated by visual activity and decreases upon visual deprivation (Hughes et al. 2018; Murphy et al. 2020). Areal differences already exist at the progenitor level because proliferation of NG2 positive OPCs can be enhanced in mouse barrel cortex after whisker lesion, but not in visual cortex after dark rearing (Mangin et al. 2012). In summary, myelination proceeded in white and gray matter and marginal zone of pig cortex well before birth with an areal-specific time course. Although myelin sheaths increased in density until P30, the basic pattern of myelination typical for the adult cortex was established at P5 in visual cortex.

## Supplementary Information

Below is the link to the electronic supplementary material.

Supplementary file1 Online Resource 1. Silver impregnation after Gallyas. A, A1. At E85 the Gallyas method detected myelin sheaths in WM, most with rather weak staining intensity. A1 shows fibers at higher magnification. Darkly impregnated blood vessels are indicated by red asterisks. B, B1. At E100, myelin was detected in WM and adjacent parts of GM. B1 shows a single impregnated axon. C, C1, C2. At P5, myelin sheaths radiated into the GM and were present in L1. D, D1. At P30, axons had fasciculated even stronger. Scale bars: 250 µm; 10 µm in the enlargements (TIF 12317 KB)

Supplementary file2 Influence of a postmortem interval on protein stability and expression of GFAP and of vimentin. A. MBP in young (P2) and adult rat cortex prepared within 30 min after death or after 24 h postmortem and storage in the cold. MBP is barely detectable in the young as expected. In the adult, MBP isoforms are present without obvious degradation after the long postmortem interval. B. GFAP is detectable in the young and stronger in the adult cortex, as expected, without obvious degradation after the long postmortem interval. C. Vimentin is detectable in the young and in the adult cortex, without obvious degradation after the long postmortem interval. D. GFAP expression increased during development to highest level at P90. The time course was rather similar in VC and SC. E. Vimentin expression was at plateau levels and constant in VC and SC from E65 to P90. Not that the developmental profiles of GFAP (increasing) and vimentin (not changing) differ from the profiles of the myelin and progenitor proteins, and with their distinct profiles also serving as a control for the blots of the oligodendrocyte markers. The numbers in or above the bars are the number of lysates (TIF 2493 KB)

## Data Availability

We confirm that all data obtained are presented in this manuscript.
