# Supplementary figures and images for: Development of myelin in fetal and postnatal neocortex of the pig, the European wild boar Sus scrofa
Source: Brain Struct Funct. 2023 Mar 31;228(3-4):947–66. doi: 10.1007/s00429-023-02633-y (PMC10147765; doi:10.1007/s00429-023-02633-y)

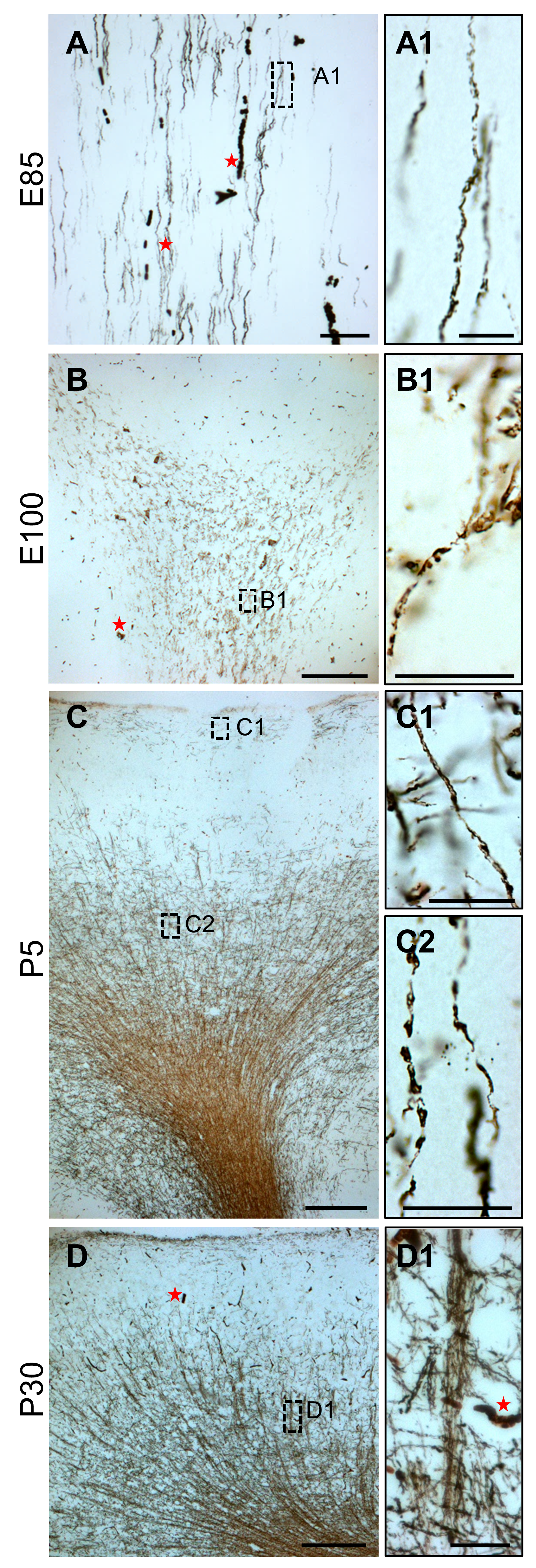

Supplement: Supplementary file 1 — Supplementary file1 Online Resource 1. Silver impregnation after Gallyas. A, A1. At E85 the Gallyas method detected myelin sheaths in WM, most with rather weak staining intensity. A1 shows fibers at higher magnification. Darkly impregnated blood vessels are indicated by red asterisks. B, B1. At E100, myelin was detected in WM and adjacent parts of GM. B1 shows a single impregnated axon. C, C1, C2. At P5, myelin sheaths radiated into the GM and were present in L1. D, D1. At P30, axons had fasciculated even stronger. Scale bars: 250 µm; 10 µm in the enlargements (TIF 12317 KB) [file 429_2023_2633_MOESM1_ESM.tif]

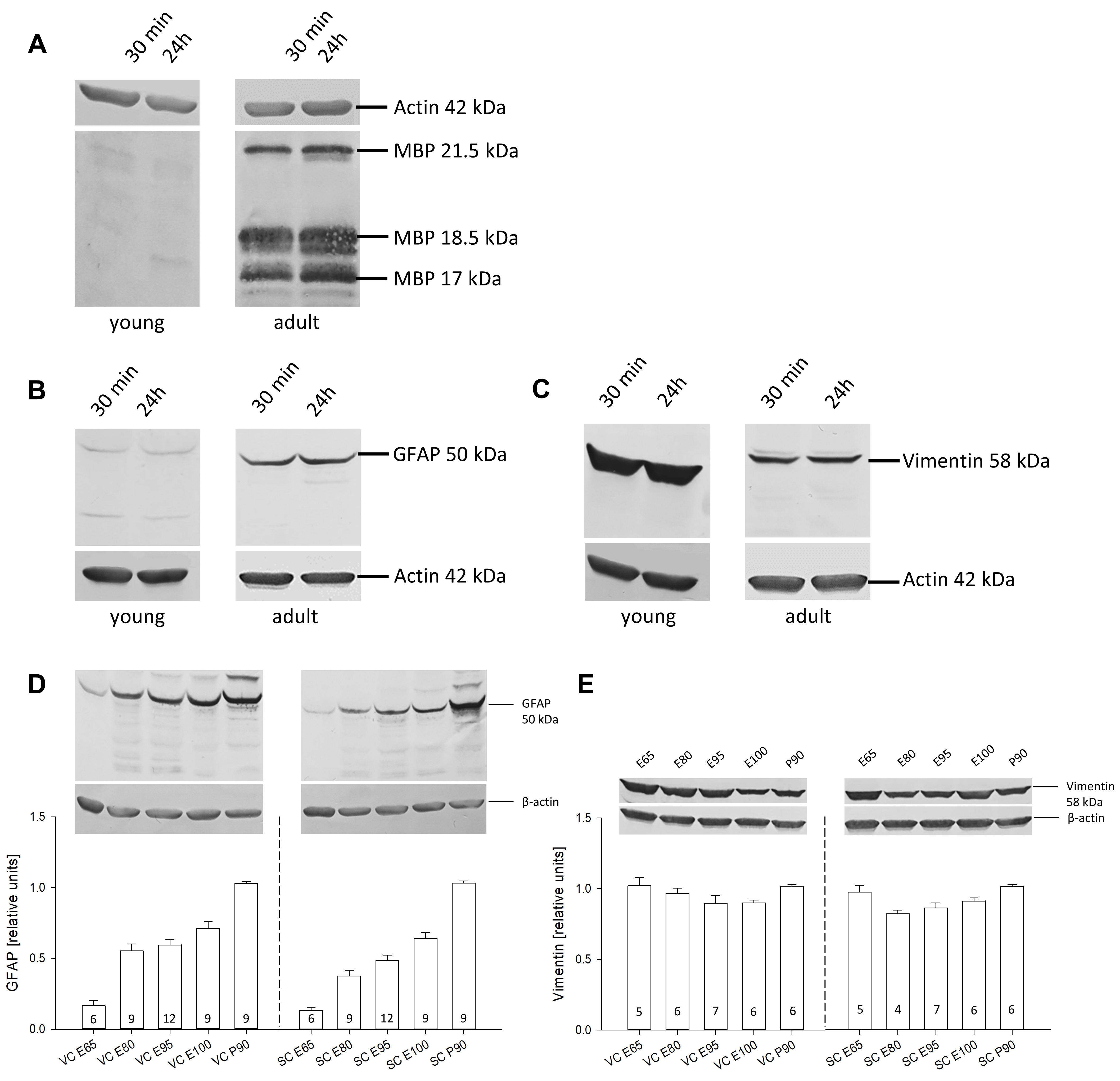

Supplement: Supplementary file 2 — Supplementary file2 Influence of a postmortem interval on protein stability and expression of GFAP and of vimentin. A. MBP in young (P2) and adult rat cortex prepared within 30 min after death or after 24 h postmortem and storage in the cold. MBP is barely detectable in the young as expected. In the adult, MBP isoforms are present without obvious degradation after the long postmortem interval. B. GFAP is detectable in the young and stronger in the adult cortex, as expected, without obvious degradation after the long postmortem interval. C. Vimentin is detectable in the young and in the adult cortex, without obvious degradation after the long postmortem interval. D. GFAP expression increased during development to highest level at P90. The time course was rather similar in VC and SC. E. Vimentin expression was at plateau levels and constant in VC and SC from E65 to P90. Not that the developmental profiles of GFAP (increasing) and vimentin (not changing) differ from the profiles of the myelin and progenitor proteins, and with their distinct profiles also serving as a control for the blots of the oligodendrocyte markers. The numbers in or above the bars are the number of lysates (TIF 2493 KB) [file 429_2023_2633_MOESM2_ESM.tif]
